# Maternal High-Fat–High-Carbohydrate Diet-Induced Obesity Is Associated with Increased Appetite in Peripubertal Male but Not Female C57Bl/6J Mice

**DOI:** 10.3390/nu12102919

**Published:** 2020-09-24

**Authors:** Debra Kulhanek, Rachel Weigel, Megan E. Paulsen

**Affiliations:** Department of Pediatrics, Division of Neonatology, University of Minnesota Medical School, Minneapolis, MN 55455, USA; kulha012@umn.edu (D.K.); rweigel@email.arizona.edu (R.W.)

**Keywords:** overnutrition, obesity, pregnancy, fetal development, hypothalamus, energy metabolism, appetite, sex differences

## Abstract

Diet-induced maternal obesity might play a critical role in altering hypothalamic development, predisposing the offspring to obesity and metabolic disease later in life. The objective of this study was to describe both phenotypic and molecular sex differences in peripubertal offspring energy homeostasis, using a mouse model of maternal obesity induced by a high-fat–high-carbohydrate (HFHC) diet. We report that males, not females, exposed to a maternal HFHC diet had increased energy intake. Males exposed to a maternal HFHC diet had a 15% increased meal size and a 46% increased frequency, compared to the control (CON) males, without a change in energy expenditure. CON and HFHC offspring did not differ in body weight, composition, or plasma metabolic profile. HFHC diet caused decreased hypothalamic glucocorticoid expression, which was further decreased in males compared to females. Maternal weight, maternal caloric intake, and male offspring meal frequency were inversely correlated with offspring hypothalamic insulin receptor (IR) expression. There was a significant interaction between maternal-diet exposure and sex in hypothalamic IR. Based on our preclinical data, we suggest that interventions focusing on normalizing maternal nutrition might be considered to attenuate nutritional influences on obesity programming and curb the continuing rise in obesity rates.

## 1. Introduction

Among the many environmental factors regulating adult onset obesity, there is a strong association between perinatal (in utero + neonatal) nutrition and adult obesity [1,2,3,4,5,6,7,8,9]. Both human studies and animal models show that fetal exposure to maternal overnutrition and obesity, with or without diabetes, is associated with sex-specific aberrancies in body weight, adiposity, hepatic function, mitochondrial function, and glucose tolerance during adulthood [1,10,11,12,13]. Programmed hyperphagia (increased energy intake) following maternal diet-induced obesity is a conserved outcome across a diverse range of experimental models, varying in model species, dietary composition, and exposure duration [6,7,14,15,16,17,18,19,20,21,22]. Collectively, these studies implicate the hypothalamus as a critical target in understanding metabolic programming, following maternal obesity. An improved understanding of hypothalamic programming might lead to the identification of modifiable signaling mechanisms, early novel interventions, and prevention of programmed obesity and metabolic disease [23,24,25,26].

Hormones, such as insulin and leptin, as well as maternal excess of glucose, lipids, and carbohydrates play a role in placental and lactational-mediated nutrient exposure to the fetus or neonate [21,26,27]. The hypothalamus is exquisitely vulnerable to injury throughout development, with the majority of development occurring by term gestation in humans (end of lactation in mice) [28]. Maternal obesity and high-fat-diet (HFD) compromise the offspring blood–brain barrier [29], stimulate proliferation of hypothalamic neuronal precursor cells and astrocytes, leading to impaired neuronal circuitry [4,26,30,31], and alter gene expression, contributing to hyperphagia [4,5,22].

Previous animal experiments largely focus on adult outcomes of hypothalamus-mediated metabolic programming, following maternal HFD-induced obesity [6,12,21]. While offspring metabolic outcomes appear to be conserved across varying diet-induced obesity models, alterations in the fat/carbohydrate ratio alters metabolic phenotypic outcomes [21]. Therefore, we used an established mouse model of maternal high-fat–high-carbohydrate (HFHC) diet-induced obesity, without diabetes, to study offspring hypothalamic phenotype [27]. An HFHC diet, as opposed to an HFD, represents a more prevalent diet composition during pregnancy [32,33,34,35,36]. Specifically, outcomes from this study are clinically relevant to 2/3 of women entering the reproductive age as overweight or obese without diabetes, in the United States [37,38]. This population is overrepresented in obstetrics and is understudied [2,39,40,41,42]. 

In this mouse model of HFHC-induced maternal obesity, we previously observed that male, but not female, HFHC offspring, gain excess weight at 8–9 weeks postnatal age on a standard chow diet [43]. At 14 weeks of age, maternal HFHC-diet-exposed male offspring, but not female offspring, show evidence of adiposity, glucose intolerance, insulin resistance, fatty liver, and cardiac dysfunction [43,44]. Neither a hypothalamic phenotype or evaluation of potential programming mechanisms at earlier timepoints in offspring, i.e., prior to the onset of weight gain and metabolic dysfunction, were evaluated in this maternal HFHC-induced obesity animal model [27,43,44,45,46,47]. 

Additionally, very few studies evaluated sex differences in offspring exposed to maternal-diet-induced obesity [1,10,11,12,13,48,49]. Previous reports mainly studied mixed-sex cohorts or studied male-only cohorts [1]. Obesity is more prevalent in women worldwide but more prevalent in men in the United States, which is suggestive of an interaction between sex and nutrient exposure [37,50]. There are established differences in growth and metabolism between sexes, which occur from conception, throughout the lifespan [51]. Reporting sex-differences might provide insight into a better understanding of mechanisms contributing to sex-specific programming, ultimately leading to sex-specific targeted therapies, to attenuate the obesity epidemic.

The objective of our study was to investigate hypothalamus-mediated energy homeostasis in offspring, following exposure to maternal HFHC diet and obesity, at a developmental time-point prior to previously observed weight gain and metabolic disease [43] We chose to study peripubertal offspring (28–40-days old), as this time point was developmentally equivalent to the hypothalamic development of term infants [3,28]. Additionally, we studied males and females separately. We hypothesized that maternal HFHC-diet-induced obesity without diabetes would cause increased energy intake in males but not females.

## 2. Materials and Methods

### 2.1. Animals and Diet

**Figure 1:**
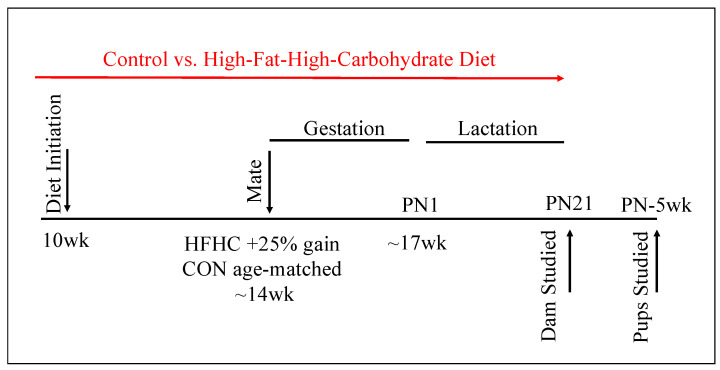
Experimental Animal Model. Female, ~10-week-old, C57Bl/6J mice were fed ad libitum, either a control or a high-fat–high-carbohydrate (HFHC) diet. Age-matched CON and HFHC females were mated once the females in the HFHC group gained 25% of their initial body weight (~14–16 weeks of age). Pregnant dams (CON N = 16, HFHC N = 15) delivered spontaneously. Litters (CON N = 16, HFHC N = 15) were culled to equal size for the lactation period. Animals were maintained on their respective diets throughout gestation and lactation. Pups were weaned at postnatal (PN) day 21 and acclimated to standard chow. Male and female pups were studied at 4–6 weeks of age. control (CON), high-fat–high-carbohydrate (HFHC).

All protocols were approved by the Institutional Animal Care and Use Committee at the University of Minnesota (protocol no. 1708-35022A). Animals were housed under controlled conditions (25 °C, 12-h light/dark cycle, lights on 0600h). Female, proven breeder, ~10 week-old, C57Bl/6J mice (The Jackson Laboratory, Bar Harbor, ME, USA) were fed ad libitum either a control (D12489B, Research Diets, New Brunswick, NJ, USA, 10.6 kcal% fat, 16.8 kcal% protein, 72.6 kcal% carbohydrate, CON) or a high-fat–high-carbohydrate (HFHC) diet (Figure 1). The HFHC diet consisted of both a high-fat pellet (Western Diet D12079B, Research Diets, New Brunswick, NJ, USA, 40 kcal% fat, 17 kcal% protein, 43 kcal% carbohydrate) and 20% sucrose solution supplemented with vitamins (AIN Vitamin Mixture, MP Biomedicals, Solon, OH, USA) and minerals (AIN-93M Mineral Mix, MP Biomedicals, Solon, OH, USA), as previously described [27]. Both fat and carbohydrate contents were increased without decreasing the protein content, in order to avoid intrauterine growth restriction. All animals had free access to water. Age-matched CON and HFHC females were mated once females in the HFHC group had gained 25% of their initial body weight (~14–16 weeks of age). Pregnant dams delivered spontaneously, to generate 16 control litters and 15 experimental litters. Litters were randomly culled to equal size for the lactation period. Animals were maintained on their respective diets throughout gestation and lactation. Mice were weighed once weekly. Weight at conception was defined as weight at time of mating. Weight at delivery was weight at gestational day (GD) 18.5, prior to spontaneous delivery of litters. Weight at weaning was defined as weight at postnatal (PN) day 21, when the litters were separated from dams. Dam’s energy consumption was determined by weighing pellets twice weekly and sucrose solution every 48 h.

Dams were studied at PN21. Offspring were studied based on maternal diet and sex (CON/Male, CON/Female, HFHC/Male, HFHC/Female). Male and female pups from each litter were weaned at PN21 and placed on standard chow. Subsets of male and female offspring were randomly allocated to experiments. A subset of animals was acclimated to the metabolic phenotyping core, where all offspring in vivo studies and necropsy were performed. Offspring were evaluated between 5–6 weeks of age. Animals were fasted for 6 h, prior to necropsy, for blood and tissue sample collection. For a particular outcome, one male or female offspring, originating from a separate litter, were randomly selected. Separate subgroups of animals were studied for separate analyses, as detailed below.

### 2.2. Collection of Tissue and Blood Samples

Dams were killed individually by CO_2_ asphyxiation and the offspring by decapitation, in the afternoon (1500 h), in a room separate from where the other animals were kept. Blood was collected within 90 s of being euthanized. Whole blood was collected at time of necropsy. Blood was obtained by cardiac puncture in adult mice and by truncal blood sampling, following decapitation in offspring mice. Whole blood glucose (mg/dL) was measured using a Contour Next glucometer (Ascensia Diabetes Care, Parsippany, NJ, USA). Blood was then spun at 5000 RPM for 10 min. Plasma (supernatant) was removed and stored at −80 °C for later analysis. The hypothalamus was dissected using the optic tract chiasm and the mammillary recess as the anterior and posterior limits, respectively, as previously described [52]. Tissue was snap frozen in liquid nitrogen and stored at −80 °C for later analysis.

### 2.3. Plasma Biochemical Analyses

Plasma cholesterol (CHOL), insulin, leptin, non-esterified fatty acids (NEFA), phospholipids (PL), triglycerides (TG), corticosterone, and adrenocorticotropic hormone (ACTH) were determined by colorimetric assay by the Mouse Metabolic Phenotyping Center at the University of Cincinnati, OH, USA. Plasma lipid profiles (TG, CHOL, PL, NEFA) were determined using specific colorimetric assays run in microtiter plates and analyzed on a plate reader. Concentrations of plasma insulin and leptin were determined using an ELISA kit from Millipore (Burlington, MA, USA). Plasma corticosterone and ACTH were measured using a corticosterone radioimmunoassay (RIA) kit and ACTH antibody (University of Minnesota, Minneapolis, MN, USA) from MP Biomedical (Salon, OH, USA).

### 2.4. In Vivo Metabolic Analyses

Glucose tolerance test (GTT) was performed by intraperitoneal injection of glucose (2 g/kg), in awake animals, after an overnight fast. Blood was collected by tail venipuncture. Body composition was determined by Echo-MRI (EchoMRI, Echo Medical Systems, Houston, TX, USA) for measurement of fat free and fat mass, as previously described [53]. Energy intake was assessed by meal pattern analysis with BioDaq (Research Diet, New Brunswick, NJ, USA), as previously described [54]. Mice were individually housed in standard cages, with individual food holders outside of the cage connected to computer-automated precision balances. Assessment of energy expenditure was performed using indirect calorimetry in single-house freely moving mice (Oxymax, Columbus Instruments, Columbus, OH, USA). The cages consisted of an indirect open circuit calorimeter that provided measurements of heat production, O_2_ consumption (VO_2_), CO_2_ production (VCO_2_), and ambulation count, during a 48-h period. Respiratory exchange ratio (RER) was determined using measured VO_2_ and VCO_2_ (Formula: RER = VCO_2_/VO_2_), as previously described [55]. Energy expenditure (EE) was determined using animal weight, RER, known constants, and VO_2_ (EE = [3.815 +1.232 × RER] × VO_2_), as previously described [53,55]. 

### 2.5. Gene Expression Analyses

Total RNA was extracted from a subset of offspring hypothalamus tissue using an RNeasy Mini Kit (Qiagen, Germantown, MD, USA). Tissues were homogenized in RLT buffer using mechanized syringe homogenization. RNA was assessed for purity and concentration using the NanoDrop (NanoDrop Technologies, Wilmington, DE, USA). Approximately 4 μg of total RNA was used to generate cDNA through reverse transcription, using a cDNA synthesis kit (Applied Biosystems, Carlsbad, CA, USA). Gene expression was assessed using pre-designed exon spanning primers (Supplementary Table S1) utilizing the TaqMan gene expressions system (Applied Biosystems, Carlsbad, CA, USA) on a QuantStudio 3 Real-Time PCR System (Applied Biosystems, Carlsbad, CA, USA). Data were normalized to 18S rRNA using the cycle threshold (ΔΔCT) method.

### 2.6. Data Presentation and Statistical Analyses

Adult female mice on a control diet or offspring of pregnant mice on a control diet were used as the control groups. In the grouped analyses, male offspring of dams on control diet were used as the control group. Experimental numbers (n/group) were determined using previous studies by our group [43], as well as expertise from the mouse phenotyping core at The University of Minnesota. Data are presented as mean ± SEM. N represents the number of animals per group. Statistical analysis and graphics were performed using the GraphPad Prism version 7 (GraphPad Software Inc., La Jolla, CA, USA). Area under the curve was calculated for both glucose tolerance tests, as a surrogate for glycemic index, as well as indirect calorimetry, as a surrogate for energy expenditure [55,56]. Results were analyzed using unpaired *t*-test to measure group differences and two-way ANOVA, followed by post-hoc Tukey’s HSD, to measure diet and sex/time effects between groups. Post-hoc was only conducted when a significant interaction between diet and sex was present in the two-way ANOVA analysis. Energy expenditure was analyzed using ANCOVA with body weight as a continuous predictor [57]. Each pup was treated as an independent subject in analyses. Pearson correlation coefficient was used as a measure of linear correlation for selected variables; *p*-value less than 0.05 was considered to be statistically significant.

## 3. Results

### 3.1. Maternal Phenotype

Metabolic phenotype characteristics of the dam cohort are outlined in Table 1. HFHC dams were 28% heavier at conception (*p* < 0.0001), 8% heavier at delivery (*p* = 0.006), and 14% heavier at weaning (*p* = 0.004) compared to the CON dams. Litter size, and male/female ratio, did not differ between groups. HFHC dams had increased energy intake during pregnancy (2.8-fold, *p* < 0.0001) and lactation (3.7-Fold, *p* < 0.0001), compared to the CON dams. Fasting glucose, insulin, triglycerides, and glucose tolerance test were performed as a measure of diabetes in the CON and HFHC dams. These markers of metabolic health did not differ between groups. HFHC dam cholesterol and leptin were increased by 32% (*p* = 0.02) and 2.5-fold (*p* < 0.0001), respectively, as compared to the CON dams. Plasma phospholipids and non-esterified fatty acids (NEFA) did not differ between the HFHC and CON dams.

### 3.2. Offspring Phenotype

**Figure 2:**
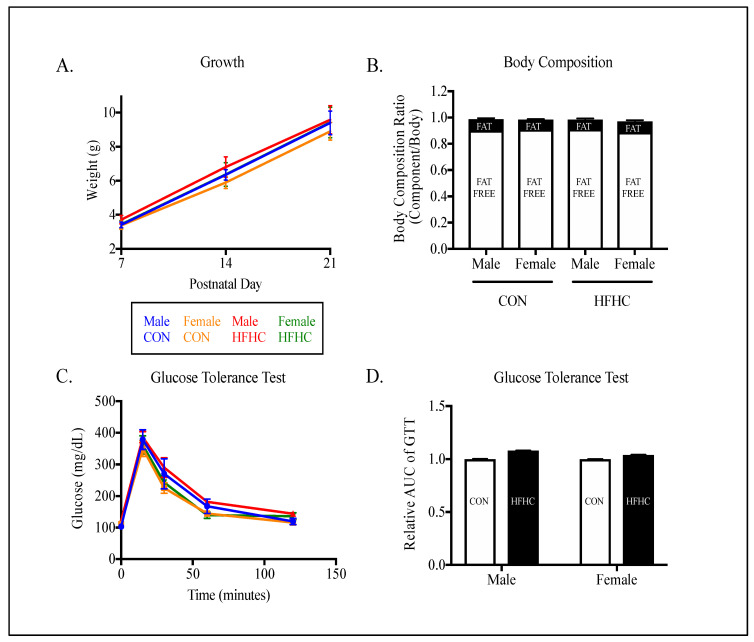
Offspring metabolic phenotype following maternal high-fat–high-carbohydrate (HFHC)-diet-induced obesity. Male and female growth was followed weekly through PN21 (**A**; *n* = 5–8/group). Body composition (% fat free mass, % fat mass, **B**; *n* = 4–6/group) and glucose tolerance test (**C**; *n* = 14–16/group) were performed post-weaning in offspring born to control (CON) and high-fat–high-carbohydrate (HFHC) dams. There were no significant differences between offspring growth (**A**), adiposity (**B**), or glucose tolerance (**D**). Results are expressed as mean ± SEM. AUC—area under the curve; unpaired *t*-test or two way ANOVA; CON—offspring born to dams maintained on control diet; HFHC—offspring born to dams maintained on high-fat–high-carbohydrate diet; blue line represents male CON offspring, red line represents male HFHC offspring, yellow line represents female CON offspring, green line represents female HFHC offspring. **B**—open bar represents % fat free mass, closed bar represents % fat mass. **D**—open bar represents CON offspring, closed bar represents HFHC offspring.

**Figure 3:**
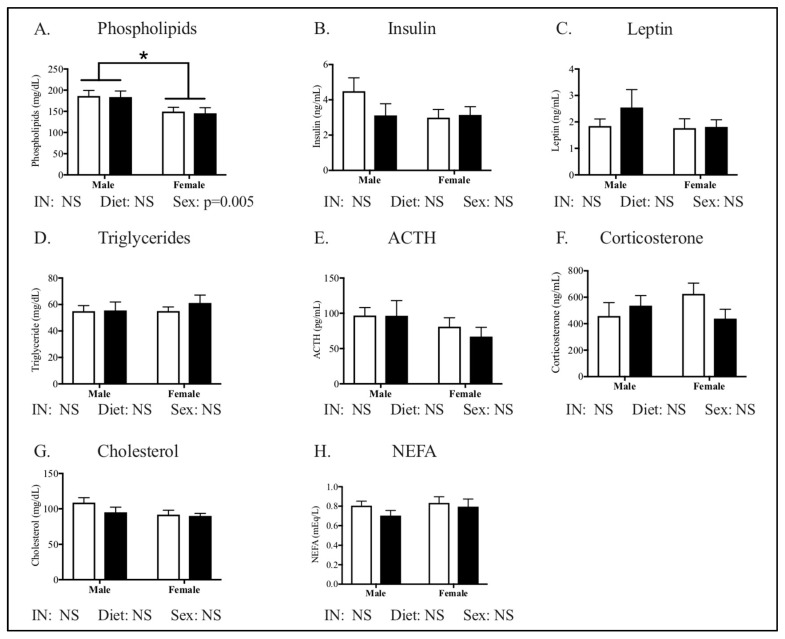
Metabolic profile in peripubertal offspring following maternal HFHC-diet-induced obesity. Plasma phospholipids (**A**), insulin (**B**), leptin (**C**), triglycerides (**D**), adrenocorticotropic hormone (**E**), corticosterone (**F**), cholesterol (**G**), and non-esterified fatty acids (**H**) were measured in fasted male and female offspring, following weaning from CON and HFHC dams. There were no significant differences between groups in either male or female offspring. Females had lower phospholipid concentrations than males (**A**, *p* = 0.005). ACTH—adrenocorticotropic hormone; NEFA—non-esterified fatty acids; CON—offspring born to dams maintained on control diet; HFHC—offspring born to dams maintained on high-fat-high-carbohydrate diet. Open bars represent CON offspring; closed bars represent HFHC offspring; Results are expressed as mean ± SEM; two-way ANOVA, *n* = 12–15 group, vs. offspring born to dams consuming same diet (diet effect) vs. male offspring born to dams consuming different diets (sex effect); * *p* < 0.05, *p* values > 0.1 were recorded as non-significant (NS).

**Figure 4:**
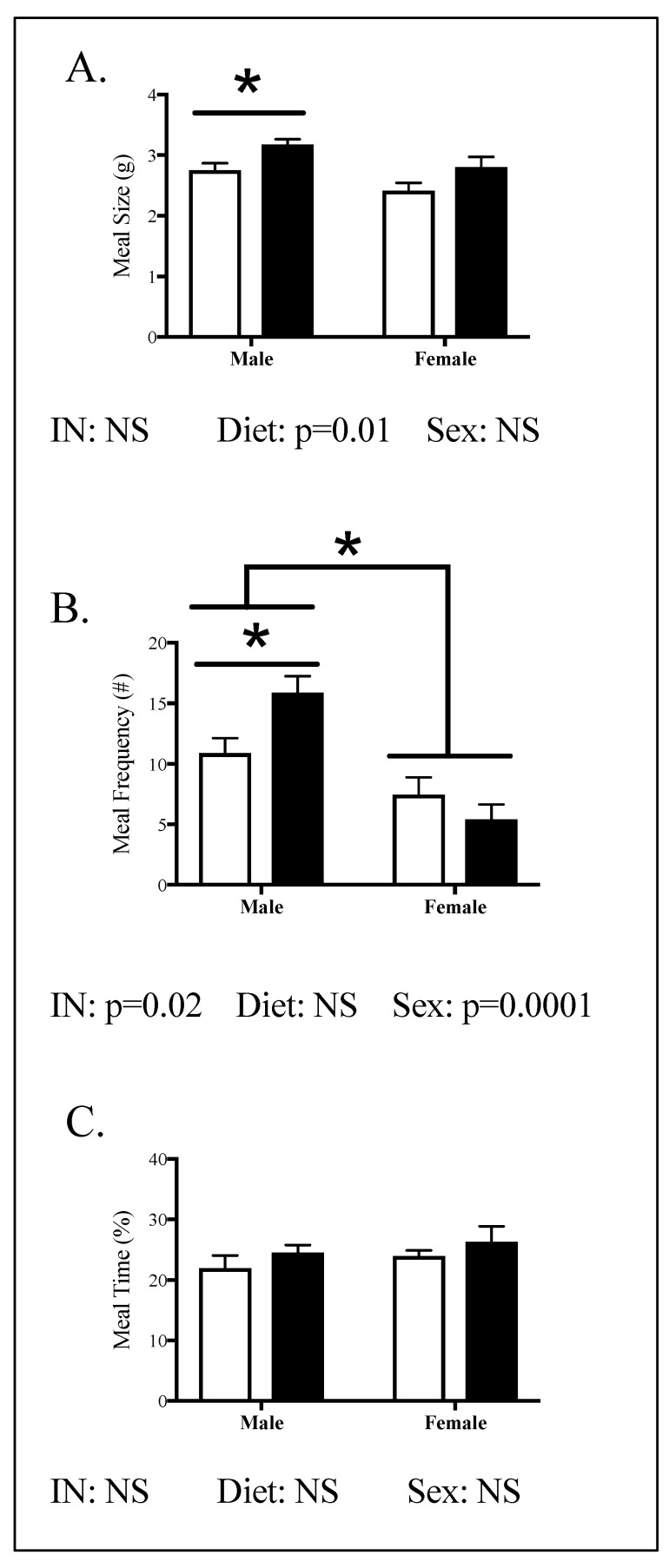
Offspring energy intake in peripubertal mice following maternal HFHC-diet-induced obesity. Meal size (**A**), meal frequency (**B**), and time spent eating (**C**) were measured in male and female offspring born to CON and HFHC dams, following weaning. HFHC males had 15% larger meals and 46% more meals than CON males (**A**,**B**). There were no significant differences between the CON and HFHC females (**A**–**C**). Male and female offspring born to HFHC dams had increased meal size, compared to CON male and female offspring (*p* = 0.01, **A**). This effect of diet was not seen in male and female offspring in meal frequency or time (**B**,**C**). Males had an increased meal frequency compared to females (*p* = 0.0001, **B**). There was an interaction between diet (CON vs. HFHC) and sex (male vs. female) in meal frequency (**B**). Results are expressed as mean ± SEM. CON—offspring born to dams maintained on control diet; HFHC—offspring born to dams maintained on high-fat–high-carbohydrate diet. Open bars represent CON offspring; closed bars represent HFHC offspring; unpaired *t*-test or two-way ANOVA, followed by post-hoc Tukey’s HSD; *n* = 4–6/group, vs. offspring born to dams consuming same diet (diet effect), vs. male offspring born to dams consuming different diets (sex effect); * *p* < 0.05, *p* values > 0.1 were recorded as non-significant (NS).

**Figure 5:**
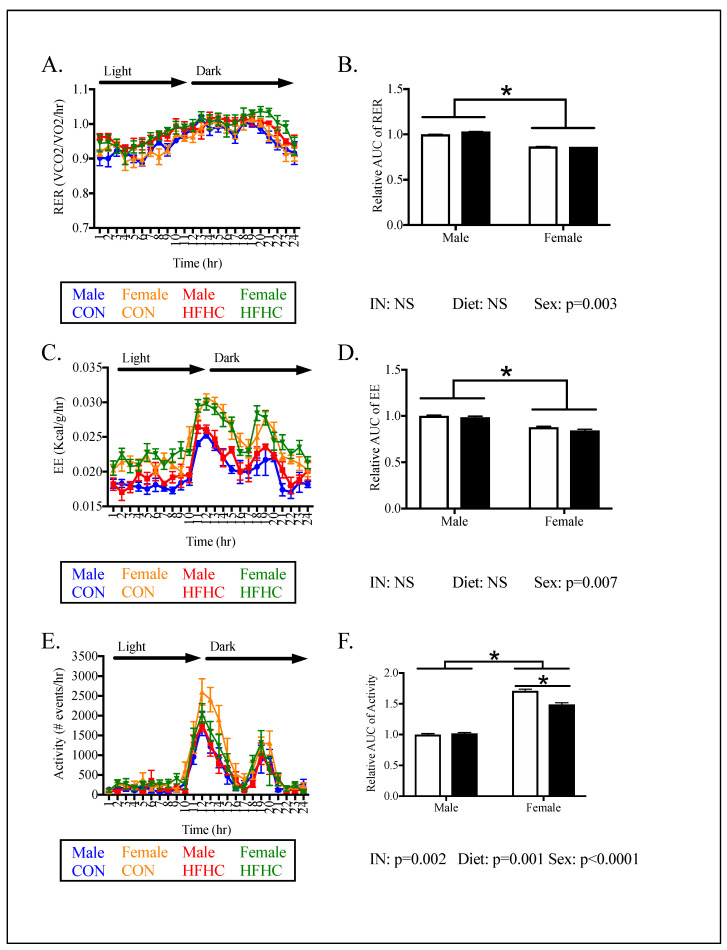
Offspring energy expenditure in peripubertal mice following maternal HFHC-diet-induced obesity. Respiratory exchange ratio (RER, **A**,**B**), energy expenditure (EE, **C**,**D**), and activity (**E**,**F**) were measured in male and female offspring born to CON and HFHC dams, following weaning. There were no differences between CON and HFHC males in energy expenditure (**B**,**D**,**F**). HFHC females did not differ in RER (**B**) or EE (Figure **D**), compared to CON groups. HFHC females showed a 13% lower activity compared to CON females (**F**). Females showed lower RER (*p* = 0.003), lower EE (*p* = 0.007), and increased activity (*p* < 0.0001), compared to males (**B**,**D**,**F**). There was an interaction between diet and sex in the analysis of activity (*p* = 0.002) but not the RER or EE measurements (**B**,**D**,**F**). Results are expressed as mean ± SEM; AUC—area under the curve; CON—offspring born to dams maintained on control diet; HFHC—offspring born to dams maintained on high-fat–high-carbohydrate diet; blue line represents male CON offspring, red line represents male HFHC offspring, yellow line represents female CON offspring, green line represents female HFHC offspring. Open bars represent CON offspring; closed bars represent HFHC offspring; unpaired *t*-test or two-way ANOVA followed by post-hoc Tukey’s HSD, *n* = 4–6/group, vs. offspring born to dams consuming the same diet (diet effect), vs. male offspring born to dams consuming different diets (sex effect); * *p* < 0.05, values > 0.1 were recorded as non-significant (NS).

Metabolic phenotype characteristics of the offspring cohort are outlined in Figure 2, Figure 3, Figure 4 and Figure 5. Offspring growth (Figure 2A), body composition (Figure 2B), and glucose tolerance (Figure 2C,D) did not differ by sex or diet. No measurements of metabolic health in plasma differed by diet between groups (Figure 3). Phospholipids were the only measurement of offspring plasma that differed by sex, with males showing increased phospholipids compared to the female offspring (Figure 3A, *p* = 0.005).

Meal pattern was assessed and the results revealed that males, not females, had increased meal size and frequency but not duration (Figure 4). Specifically, male HFHC offspring showed 15% increased meal size (Figure 4A, *p* = 0.02) and 46% increased frequency (Figure 4B, *p* = 0.03), compared to male CON offspring. Time spent eating did not differ between CON and HFHC males (Figure 4C). There was a significant effect of HFHC diet on meal size (*p* = 0.01, Figure 4A) but not meal number or time in either males or females (Figure 4). Females ate fewer meals per day compared to males in both CON and HFHC groups (Figure 4B). Neither meal size or meal time was significantly different between males and females (Figure 4). There was an interaction between diet and sex in meal number (*p* = 0.02) but not meal size or meal time.

Energy expenditure (EE) was determined by indirect calorimetry and home cage activity (Figure 5). In general, the analysis of energy expenditure did not reveal differences between HFHC and controls (Figure 5). However, there were sex differences in energy expenditure, with females demonstrating lower RER and EE but higher activity, when compared to males (Figure 5). HFHC females had 13% lower activity compared to CON females (Figure 5F).

### 3.3. Offspring Hypothalamic Appetite-Regulating Gene Expression

**Figure 6:**
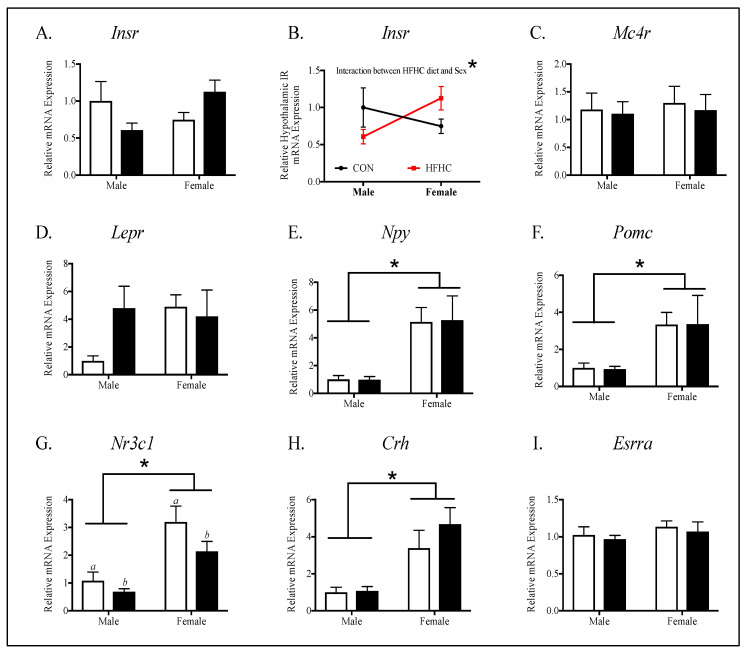
Offspring hypothalamic appetite signaling in peripubertal mice, following maternal HFHC-diet-induced obesity. Hypothalamic gene expression of insulin receptor (**A**), melanocortin 4 receptor (**C**), leptin receptor (**D**), neuropeptide-Y (gene transcript for hunger neuropeptide, **E**), proopiomelanocortin (gene transcript for satiety neuropeptide, **F**), glucocorticoid receptor (**G**), corticotropin-releasing hormone (**H**), and estrogen receptor alpha (**I**) were measured in fasted offspring, following weaning from CON and HFHC dams. There were no significant differences between CON and HFHC groups in the male or female offspring studied separately. There was an interaction between diet and sex in insulin receptor (**B**) such that males had a lower insulin receptor expression and the females had higher insulin receptor expression, when exposed to maternal HFHC diet (**B**, *p* = 0.03). The HFHC offspring had a lower glucocorticoid receptor expression (**C**, *p* = 0.05) compared to the CON offspring. The female offspring had a higher glucocorticoid receptor (**C**, *p* < 0.0001), NPY (**D**, *p* < 0.001), POMC (**E**, *p* = 0.001), and CRH (**F**, *p* = 0.004), compared to the male offspring. Results are expressed as mean ± SEM. CON—offspring born to dams maintained on control diet; HFHC—offspring born to dams maintained on high-fat–high-carbohydrate diet; black line (**B**) represents maternal CON-diet-exposed offspring; red line (**B**) represents maternal HFHC-diet-exposed offspring. NPY—neuropeptide-Y; POMC—proopiomelanocortin; CRH—corticotropin-releasing hormone. Open bars represent CON offspring; closed bars represent HFHC offspring; unpaired *t*-test or two-way ANOVA, followed by post-hoc Tukey’s HSD, *n* = 7–13/group, vs. offspring born to dams consuming same diet (diet effect), vs. male offspring born to dams consuming different diets (sex effect); * *p* < 0.05, *p* values > 0.1 were recorded as non-significant (NS).

Next, to determine potential etiologies for sex-specific differences in energy homeostasis, we evaluated key targets involved in appetite signaling in offspring hypothalamus (Figure 6). There were no differences between the CON and HFHC groups in the expression of the gene targets measured in males and female offspring. Females had higher neuropeptide-y (NPY) (Figure 6E, *p* < 0.001), proopiomelanocortin (POMC) (Figure 6F, *p* = 0.001), glucocorticoid receptor (GR) (Figure 6G, *p* < 0.0001), and corticotropin-releasing hormone (CRH) (Figure 6H, *p* = 0.0004), when compared to males. HFHC males and females had lower hypothalamic GR than CON males and females (Figure 6G, *p* = 0.05). There was a significant interaction between sex and diet in the analysis of insulin receptor (IR) (Figure 6B, *p* = 0.03).

### 3.4. Relationships between Phenotype and Energy Homeostasis

**Figure 7:**
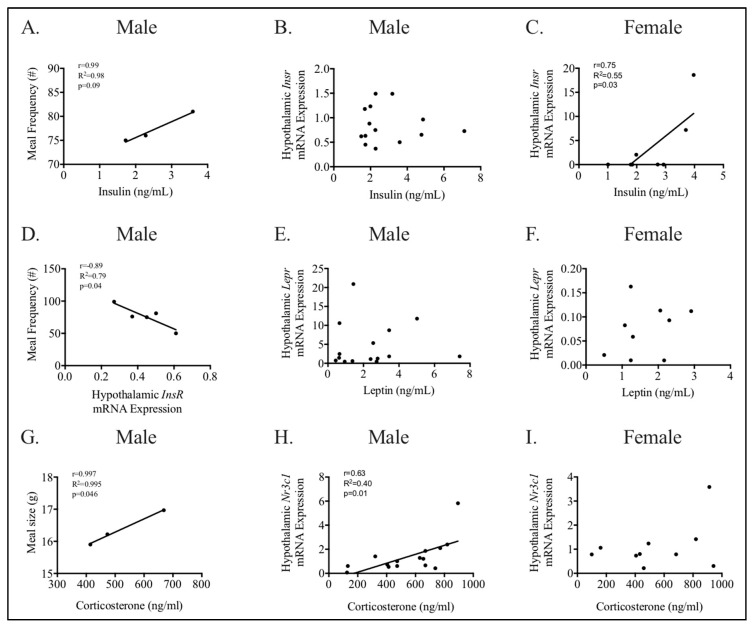
Relationships between appetite, peripheral hormones, and hypothalamic receptors are different between peripubertal males and females. Appetite was measured using metabolic chambers to determine meal size and frequency (**A**,**D**,**G**). Male meal frequency correlated with hypothalamic mRNA expression of insulin receptor (**D**) and trended towards correlation with fasted plasma insulin (**A**). Fasted male plasma corticosterone was directly and strongly correlated with meal size (**G**). Relationships between peripheral hormones and respective hypothalamic receptors involved in appetite were determined in males (**B**,**F**,**H**) and females (**C**,**F**,**I**). Male corticosterone and hypothalamic glucocorticoid (*Nr3c1*) mRNA expression were strongly and directly correlated (**H**). Fasted male insulin (**B**) and leptin (**E**) did not correlate with mRNA expression of their respective hypothalamic receptors. Female fasted plasma insulin directly and strongly correlated with mRNA expression of the hypothalamic insulin receptor (**C**). Fasted female leptin (**F**) and corticosterone (**I**) did not correlate with mRNA expression of their respective hypothalamic receptors. Results are expressed as correlation coefficient (*r*) and linear regression (R^2^); *p* < 0.05 was considered significant; *n* = 3–5 (**A**,**C**,**G**), *n* = 13–15 (**B**,**E**,**H**); *n* = 7–10 (**C**,**F**,**I**); *Insr*—insulin receptor; *Lepr*—leptin receptor, and *Nr3c1*—glucocorticoid receptor.

To determine potential etiologies for increased energy intake in male HFHC compared to male CON offspring, we performed correlation analysis between aspects of maternal and offspring energy homeostasis phenotype (Figure 7 and Supplementary Tables S2 and S3). Maternal weight, consumption, and hormonal milieu were not associated with energy intake (Supplementary Table S2). Maternal weight at conception (*r* = −0.57, *p* = 0.04) but not delivery, and consumption during pregnancy (*r* = −0.56, *p* = 0.04) and lactation (*r* = −0.54, *p* = 0.048) were moderately and inversely correlated with male IR mRNA expression in the hypothalamus (Supplementary Table S2). Conversely, maternal weight at delivery (*r* = 0.56, *p* = 0.04) but not conception, and consumption during pregnancy (*r* = 0.55, *p* = 0.04) but not lactation, were moderately and directly correlated with male leptin receptor (LR) mRNA expression in the hypothalamus (Supplementary Table S2). Maternal weight at delivery was associated with NPY (hunger neuropeptide) mRNA expression in the hypothalamus (*r* = −0.58, *p* = 0.04). No other relationships between maternal weight, consumption, or hormonal milieu, and hypothalamic receptor (insulin, leptin, glucocorticoid receptors) or neuropeptide (POMC, NPY) mRNA expression were found (Supplementary Table S2).

Next, we performed correlation analysis between hypothalamic receptor (insulin, leptin, glucocorticoid receptors) mRNA expression, neuropeptide (POMC, NPY) mRNA expression, and peripheral hormone concentrations with energy intake and expenditure, to determine a potential etiology for increased meal size/frequency in HFHC-exposed males (Figure 7, Supplementary Table S3). Male plasma corticosterone, but not hypothalamic GR, was strongly and directly correlated to meal size (Figure 7G, Supplementary Table S3, *r* = 0.99, *p* = 0.046). Male hypothalamic IR mRNA expression was strongly and inversely correlated to meal frequency (Figure 7D, *r* = −0.89, *p* = 0.04), and the plasma insulin trended towards being correlated with meal frequency (Figure 7A, *r* = 0.99, *p* = 0.09) (Supplementary Table S3). Hypothalamic mRNA expression of LR, POMC, NPY, or plasma leptin were not associated with meal size or frequency (Supplementary Table S3).

As we found sex-specific differences in hypothalamic insulin and GR mRNA expression, we investigated the correlations between respective peripheral hormones and hypothalamic receptors in male and females (Figure 7). In males, plasma corticosterone was directly and moderately correlated with hypothalamic GR mRNA expression (*r* = 0.63, *p* = 0.01, Figure 7H). Conversely, both plasma insulin and leptin did not correlate with the mRNA expression of their respective hypothalamic receptors (Figure 7B,E). In females, the plasma insulin directly and strongly correlated with hypothalamic IR mRNA expression (*r* = 0.75, *p* = 0.03, Figure 7C). However, both plasma leptin and corticosterone did not correlate with mRNA expression of their respective hypothalamic receptors (Figure 7F,I).

## 4. Discussion

The objective of this study was to investigate early hypothalamic programming in males and females exposed to maternal HFHC-induced obesity, in an established mouse model. We found that male mice exposed to maternal HFHC-induced obesity ate larger meals more often, without a compensatory change in energy expenditure. If this energy imbalance (energy intake > energy expenditure) were to persist, HFHC males would show an excessive weight-gain, compared to CON male mice. Female mice did not show differences between energy intake and overall energy expenditure between groups. Both CON and HFHC males and females did not differ in growth, body composition, or glucose tolerance.

Previous studies using this mouse model of HFHC-induced obesity in pregnancy showed significant differences in weight-gain between HFHC and CON males beginning at 8–9 weeks, and no changes in female weight through 14 weeks, postnatally [43]. Assuming a similar phenotype in this cohort of male mice, this study demonstrated an increase in male energy intake preceding changes in body weight, adiposity, and glucose homeostasis. Early intervention in disordered eating habits, such as those seen in our HFHC-exposed males, might prevent obesity and improve cardiometabolic health [58,59,60,61]. Pre-clinical studies showed that regular meal patterns with intermittent energy restriction periods could improve metabolic health and reverse obesity and cardiovascular disease [60,61]. Future studies investigating regulated meal patterns, an easily operationalized intervention, might provide a novel intervention in attenuating obesity and metabolic programming, following exposure to maternal HFHC-diet induced obesity.

### 4.1. The Effect of Maternal HFHC-Diet-Induced Obesity on Energy Homeostasis

HFHC-diet induced maternal obesity was not directly correlated with energy intake, as hypothesized. Male hypothalamic IR mRNA expression and fasting plasma corticosterone concentration, however, were strongly correlated with energy intake. Maternal weight at conception and caloric intake were moderately and inversely correlated with male hypothalamic IR mRNA expression, but not male corticosterone concentration. Male corticosterone was moderately and directly correlated with hypothalamic GR mRNA expression. Hypothalamic GR mRNA expression was decreased in male HFHC mice but was not associated with maternal weight or caloric intake. We did not find diet-induced effects on neuropeptides NPY (hunger) and POMC (satiety). Additionally, we did not find differences in hypothalamic LR expression between groups. We speculate that HFHC-diet induced maternal obesity might directly cause changes to hypothalamic IR expression, whereas decreased GR expression might be a secondary effect of HFHC-diet-induced maternal obesity.

Insulin receptors are densely expressed in the arcuate nucleus (ARC) of the hypothalamus, which is the primary site of appetite regulation [7]. Activation of IRs decrease NPY and increase POMC expression and action in the ARC [23,62,63]. Previous studies evaluating transgenic mice with decreases in neuronal IRs led to hyperphagia and obesity [64,65]. Less hypothalamic insulin activation, as implicated in an indirect correlation with meal frequency in our study, decreases the synaptic input to the periventricular nucleus (PVN), thereby, decreasing input to the melanocortin system [62,66]. Further investigation of hypothalamus-mediated insulin signaling following exposure to maternal HFHC-induced obesity at serial developmental time-points would further determine the role that hypothalamic IR plays in programming of obesity.

Glucocorticoid receptors are expressed throughout the hypothalamus, including the ARC and PVN. GR, in the ARC of the hypothalamus, is involved in the regulation of energy homeostasis, through expression of neuropeptides NPY and POMC [5]. A previous study investigating hypothalamic GR mRNA expression in sheep, following maternal undernutrition, showed increased GR mRNA expression in fetal hypothalamus, which persisted up to 5 years in offspring [67]. Other pre-clinical studies showed conflicting results with GR activation, both increasing or decreasing the POMC neuropeptide expression [5].

Decreased GR expression in the PVN of the hypothalamus, however, consistently show GR to be an important regulator of negative feedback in the hypothalamic–pituitary–adrenal (HPA) axis [68,69,70]. Previous studies show that loss of GR function in adult mice causes HPA axis overactivity, hypothalamic growth impairment, and peripheral metabolic compromise in adolescent and adult mice [68,70]. One study demonstrated that severe decreased PVN GR (87% below control PVN), induced by genetic modification, is associated with obesity and hyperglycemia, emerging at 3 months of age in adult mice [70]. Further investigation of hypothalamic GR expression, GR-mediated neuropeptide expression, and HPA-axis activation at later developmental time-points would clarify the programming role of hypothalamic GR in the development of obesity, following exposure to HFHC-induced maternal obesity.

We did not find increased NPY or decreased POMC neuropeptide expression in our study, as others have reported [7,22,71,72,73]. It is possible that maternal obesity and overnutrition influenced the hypothalamic synaptic inputs of NPY and POMC neurons, which we did not investigate, rather than neuropeptide expression of NPY and POMC, which we did report. Additionally, it is plausible that the study of our offspring in the fasted state, rather than the fed state, influenced neuropeptide expression. Previous studies reported that NPY is downregulated in offspring of obese dams to control levels when in the fasted state, as compared to the fed state [74]. Additionally, we evaluated whole hypothalamic homogenates rather than separate hypothalamic nuclei (i.e., ARC or PVN), which might have influenced our results. Lastly, other studies differed in macronutrient diet content (high-fat–low-carbohydrate diet vs. high-fat–high-carbohydrate) or maternal glucose tolerance (diabetic vs. nondiabetic), which might have influenced differences between our results and others. Future studies evaluating hypothalamic morphology and function might further characterize mechanisms between decreased receptor expression and hyperphagia.

Additionally, we did not find associations between elevated maternal leptin and offspring energy intake, fasted plasma leptin, or hypothalamic mRNA expression of LR. Normal regulation of leptin during critical windows of development is essential for the normal development of the hypothalamus and appetite signaling [21,75,76,77,78]. Leptin, like insulin, is neurotrophic and when aberrant, could lead to altered hypothalamic morphology and function. Additionally, leptin inhibits NPY and activates POMC, creating a negative feedback loop that prevents obesity. Early alterations to this feedback loop were thought to contribute to the programming of obesity [21]. We might not have found maternal HFHC-induced changes in plasma or hypothalamic leptin because the HFHC offspring did not show increased adiposity. We did find that plasma leptin and hypothalamic LR mRNA expression were not correlated in male or female offspring, potentially demonstrating an early alteration in the central leptin feedback loop. Further studies at later developmental time-points evaluating the relationship between plasma leptin and hypothalamic LR function, following maternal HFHC-induced obesity would help clarify the role of leptin in programming obesity.

### 4.2. Sex-Specific Effects of Maternal HFHC-Diet-Induced Obesity on Energy Homeostasis

Males showed an increased energy intake, compared to females, consistent with our hypothesis. The majority of studies using animal models of maternal obesity and overnutrition reported results of mixed sex cohorts or focused only on male offspring results [1]. Studies that evaluated sex differences in offspring exposed to maternal obesity and overnutrition showed that males were more susceptible to changes in body weight and adiposity, whereas females were more susceptible to changes in glucose homeostasis [79,80,81]. Although investigation of the mechanisms contributing to sex-specific differences in offspring energy homeostasis were beyond the scope of this study; potential underlying mechanisms include a complex interplay between sex chromosomes and hormones [82].

Sex differences were demonstrated in GR, corticotropin releasing hormone (CRH), and IR hypothalamic mRNA expression. There were significant decreases in hypothalamic GR mRNA expression in males (vs. females) and mice exposed to an HFHC diet (vs. CON diet). CRH was only affected by sex, with females demonstrating increased mRNA expression compared to males. Increased female hypothalamic CRH and GR mRNA expression was suggestive of increased baseline hypothalamic activity of the hypothalamic–pituitary–adrenal (HPA) axis, compared to males. Additionally, males but not females, had a direct correlation between plasma corticosterone and hypothalamic mRNA expression of GR, which might indicate alterations in the feedback loop associated with the female HPA-axis.

PVN GR-mediated negative feedback occurs by adolescence in male mice and early adulthood in female mice [68,70]. It is possible that both diet and sex-specific changes to hypothalamic GR might proceed alterations in the HPA-axis feedback and not be related to appetite, until later developmental time-points. Early increased hypothalamic GR in females, as demonstrated in our study, in addition to reported later function of the HPA-axis feedback in females by others, might contribute to males developing obesity earlier than female offspring when exposed to maternal obesity.

Neither diet nor sex had independent effects on hypothalamic IR mRNA expression, however, there was an interaction between diet and sex, such that males exposed to HFHC diet had decreased IR expression and females exposed to HFHC diet had increased IR expression. As hypothalamic IR expression is directly associated with meal frequency, this might explain why females are protected from increased energy intake at this developmental time-point. Additionally, females, not males, had a direct correlation between plasma insulin and hypothalamic mRNA expression of IR, which might indicate alterations in the insulin feedback loop associated with male insulin signaling. Our results are consistent with a previous study of maternal HFD-induced diabetes in pregnancy, demonstrating offspring adiposity and glucose intolerance at 130 days of life, in both males and females. Hypothalamic ARC mRNA IR expression was decreased in HFD-exposed male offspring and trended towards an increase in HFD-exposed female offspring, similar to our study [83]. This study, however, did not evaluate for an interaction between HFD and sex, in contrast to our study.

There are limitations to consider in the interpretation of our study results. Our study did not evaluate hypothalamic morphology, synaptic function, differences between hypothalamic nuclei, or consider an exhaustive evaluation of appetite regulation targets in offspring exposed to maternal obesity, all of which might contribute to programming of obesity. Correlations between energy homeostasis and maternal/offspring metabolic status and hypothalamic function were limited by small sample sizes. As we were able to report strong correlations with small sample sizes, our study might be underpowered to detect all relationships. Although there are established sex-specific influences in early organ development and placental response to HFHC maternal obesity [1,49,84], evaluation of sex differences prior to puberty in mice might miss sex-differences that emerge during later developmental time-points. Lastly, our study evaluated a single time-point rather than several ones, thus, making it difficult to know definitively that the cohort studied here would have developed obesity and metabolic disease in adulthood, as speculated in our findings, based on previous studies.

## 5. Conclusions

Our study is clinically relevant to appetite programming in offspring born to obese mothers without diabetes who were consuming a high-fat–high-carbohydrate diet. We concluded that the maternal HFHC-diet-induced obesity caused male but not female offspring, to show increased food intake, and both male and female offspring to show decreased hypothalamic GR mRNA expression. Maternal weight and caloric intake were associated with hypothalamic insulin receptor expression, which was differentially expressed in male and females exposed to an HFHC diet. Preconception and prenatal interventions focusing on maternal nutrition, in addition to postnatal interventions, targeting feeding behaviors, and hypothalamic appetite signaling during critical windows of development, might attenuate nutritional influences on obesity programming.

## Figures and Tables

**Table 1:** Dam Cohort Characteristics.

	CON	HFHC	*p* Value
***Pregnancy***			
Body weight (conception), g	26.17 ± 0.40	33.51 ± 0.41	<0.0001
Body weight (delivery), g	38.38 ± 0.80	41.63 ± 0.64	0.006
Body weight (weaning), g	26.87 ± 0.40	30.68 ± 1.40	0.004
Litter size, #	7.0 ± 0.5	6.0 ± 0.4	NS
Male offspring/litter, %	54 ± 4.4	43 ± 5.8	NS
***Consumption***			
Energy intake (pregnancy), kcal/day	21.17 ± 6.07	59.87 ± 3.61	<0.0001
Energy intake (lactation), kcal/day	27.17 ± 0.88	99.80 ± 7.05	<0.0001
***Metabolic***			
Glucose, mg/dL	89.4 ± 3.6	83.7 ± 4.0	NS
Glucose tolerance test, AUC	25.43 ± 1.94	22.22 ± 1.16	NS
Cholesterol, mg/dL	138.3 ± 16.5	182.3 ± 9.7	0.02
Insulin, ng/mL	1.62 ± 0.5	2.05 ± 0.2	NS
Leptin, ng/dL	4.13 ± 0.49	10.2 ± 0.9	<0.0001
NEFA, mEq/L	0.74 ± 0.05	0.83 ± 0.04	NS
Phospholipids, mg/dL	209.8 ± 15.1	239.1 ± 12.4	NS
Triglycerides, mg/dL	63.4 ± 5.7	69.8 ± 4.1	NS

Results are expressed as mean ± SEM, *p* values > 0.1 recorded as nonsignificant (NS). Maternal metabolic characteristics obtained at weaning, *n* = 16/CON group, *n* = 15/HFHC group. Group differences measured by unpaired *t*-test. control (CON), high-fat–high-carbohydrate (HFHC).

## Supplementary Materials

The following are available online at https://www.mdpi.com/2072-6643/12/10/2919/s1. Table S1: RT-qPCR Gene Targets. Table S2: Maternal Relationship with Male Offspring Energy Homeostasis. Table S3: Offspring Energy Homeostasis Relationship with Hypothalamic Appetite Signaling.
