# Supplementary material for: Maternal High-Fat–High-Carbohydrate Diet-Induced Obesity Is Associated with Increased Appetite in Peripubertal Male but Not Female C57Bl/6J Mice
Source: Nutrients. 2020 Sep 24;12(10):2919. doi: 10.3390/nu12102919 (PMC7598591; doi:10.3390/nu12102919)
Supplement: Supplementary file 1 [file nutrients-12-02919-s001.pdf]

Supplemental Table 1. RT-qPCR Gene Targets

Gene Name	Gene Symbol	Assay ID	Catalog #
corticotropin releasing hormone	*Crh*	Mm04206019_m1	4331182
estrogen related receptor alpha	*Esrra*	Mm00433143_m1	4331182
insulin receptor	*Insr*	Mm01211875_m1	4331182
leptin receptor	*Lepr*	Mm00440181_m1	4331182
melanocortin 4 receptor	*Mc4r*	Mm00457483_s1	4331182
neuropeptide Y	*Npy*	Mm01410146_m1	4331182
nuclear receptor subfamily 3, group C, member 1	*Nr3c1*	Mm00433832_m1	4331182
pro-opiomelanocortin-alpha	*Pomc*	Mm00435874_m1	4331182

TaqMan gene expression assays purchased from ThermoFisher Scientific, Carlsbad, CA.

Supplemental Table 2. Maternal Relationship with Male Offspring Energy Homeostasis

	Meal Size (g)	Meal Frequency (#)	Energy Expenditure	Hypothalamic *Insr*	Hypothalamic *Lepr*	Hypothalamic *Nr3c1*	Hypothalamic *Pomc*	Hypothalamic *Npy*
**Pregnancy**								
Body weight (conception), g	NS	NS	NS	(-) 0.57**	NS	NS	NS	NS
Body weight (delivery), g	NS	NS	(+) 0.94*	NS	(+) 0.56**	NS	NS	(-) 0.58**
**Consumption**								
Energy intake (pregnancy), kcal/day	NS	NS	(+) 0.94*	(-) 0.56**	(+) 0.55**	NS	NS	NS
Energy intake (lactation), kcal/day	NS	NS	(+) 0.94*	(-) 0.54**	NS	NS	NS	NS
**Metabolic**								
Cholesterol, mg/dL	NS	NS	NS	NS	NS	NS	NS	NS
Insulin, ng/mL	NS	NS	NS	NS	NS	NS	NS	NS
Leptin, ng/dL	NS	NS	NS	NS	(+) 0.46*	NS	(-) 0.46*	NS
NEFA, mEq/L	NS	NS	NS	NS	NS	NS	NS	NS
Phospholipids, mg/dL	NS	NS	NS	NS	NS	NS	NS	NS
Triglycerides, mg/dL	NS	NS	NS	NS	NS	NS	NS	NS

Results are expressed as correlation coefficient (R), ** p<0.05, *p=0.05-0.09, p>0.1 recorded as nonsignificant (NS). NEFA: nonesterfied fatty acids; *Insr* : mRNA expression of hypothaamic insulin receptor; *Lepr:* mRNA expression of hypothaamic leptin receptor; *Nr3c1* : mRNA expression of hypothaamic glucocorticoid receptor; *Pomc:* mRNA expression of hypothaamic proopiomelanocortin (satiety neuropeptide); *Npy* : mRNA expression of hypothaamic neuropeptide y (hunger neuropeptide). N=3-5 (meal size, meal frequency, and energy expenditure); N=13-16 (*Insr, Lepr, Nr3c1, Pomc, Npy*).

Supplemental Table 3. Offspring Energy Homeostasis Relationship with Hypothalamic Appetite Signaling

	Meal Size (g)	Meal	Energy
**Appetite Hypothalamic Receptor**			
Hypothalamic *Insr*	NS	(-) 0.89**	NS
Hypothalamic *Lepr*	NS	NS	NS
Hypothalamic *Nr3c1*	NS	NS	NS
**Appetite Neuropeptide**			
Hypothalamic *Pomc*	NS	NS	NS
Hypothalamic *Npy*	NS	NS	NS
**Peripheral Hormone**			
Insulin, ng/mL	NS	(+) 0.99*	NS
Leptin, ng/dL	NS	NS	NS
Corticosterone, ng/dL	(+) 0.99**	NS	NS
NEFA, mEq/L	NS	NS	(-) 0.99*
Phospholipids, mg/dL	NS	NS	(+) 0.99**

Results are expressed as correlation coefficient (R), ** p<0.05, *p=0.05-0.09, p>0.1 recorded as nonsignificant (NS). NEFA: nonesterfied fatty acids; *Insr* : mRNA expression of hypothaamic insulin receptor; *Lepr:* mRNA expression of hypothaamic leptin receptor; *Nr3c1* : mRNA expression of hypothaamic glucocorticoid receptor; *Pomc:* mRNA expression of hypothaamic proopiomelanocortin (satiety neuropeptide); *Npy* : mRNA expression of hypothaamic neuropeptide y (hunger neuropeptide). N=3-5.
